# Einzelhandel als Katalysator für nachhaltige urbane Radlogistik? – WüLivery, ein Fallbeispiel aus Würzburg

**DOI:** 10.1007/s00548-021-00758-y

**Published:** 2021-12-07

**Authors:** Alexandra Appel, Sina Hardaker

**Affiliations:** Institut für Geographie, Lehrstuhl für Wirtschaftsgeographie, JMU Würzburg, Am Hubland, 97074 Würzburg, Deutschland

**Keywords:** Urbane Logistik, Nachhaltigkeitstransformation, Letzte Meile, Einzelhandel, Lokaler Onlinemarktplatz, Urban logistics, Sustainability transition, Last Mile, Retail, Local Online marketplace

## Abstract

Die Covid-19-Pandemie gilt in vielen gesellschaftlichen Teilbereichen als Beschleuniger für Transformationsprozesse. Auch im Bereich der Organisation urbaner Logistik und Einzelhandelslandschaften etablieren sich neue Akteur*innen und Funktionen. Logistiker*innen integrieren lokale Onlinemarktplätze in ihre Profile und der stationäre Einzelhandel generiert Wettbewerbsfähigkeit gegenüber großen Onlinehändler*innen über die Nutzung lokaler Radlogistiknetzwerke, mittels derer Lieferungen noch am Tag der Bestellung (Same-Day-Delivery) verteilt werden können. Damit leisten die involvierten Akteur*innen potenziell auch einen Beitrag zur Nachhaltigkeitstransformation im Bereich urbaner Logistiksysteme. Im Fokus steht das Fallbeispiel *WüLivery*, ein Kooperationsprojekt des Stadtmarketingvereins, der Wirtschaftsförderung, Radlogistiker*innen sowie Einzelhändler*innen in Würzburg, welches während des zweiten coronabedingten Lockdowns im November 2020 umgesetzt wurde. Die entstehenden Dynamiken und Organisationsformen werden auf Basis von 11 Expert*inneninterviews dargestellt und analysiert. Es kann gezeigt werden, dass städtische Akteur*innen grundlegende Mediator*innen für Transformationsprozesse darstellen und Einzelhändler*innen und lokale Onlinemarktplätze als Katalysator*innen fungieren können. Das ist auch vor dem Hintergrund planerischer und politischer Kommunikationsprozesse zur Legitimation neuer Verkehrsinfrastrukturen nutzbar, da die einzelnen Akteur*innengruppen in Austausch kommen und ein gesteigertes Bewusstsein für die jeweiligen Bedarfe entsteht.

## Abstract (en)

The Covid-19 pandemic is seen in many parts of society as a catalyst for transformation processes. In the field of urban logistics organization and retail landscapes, too, new stakeholders and functions are becoming established. Logistics companies are integrating local online marketplaces into their profiles and brick-and-mortar retailers are generating the ability to compete against large online retailers using bicycle logistics networks, by which deliveries can be distributed on the day of ordering (same-day delivery). In this way, the stakeholders involved potentially also make a contribution to the transformation of sustainability in the field of urban logistics systems. The focus is on the example of *WüLivery*, a collaborative project of the city marketing association, the business development department, bicycle logistics specialists and retailers in Würzburg, that was implemented during the second coronavirus-related lockdown in November 2020. The existing dynamics and forms of organization are presented and analyzed based on 11 expert interviews. It can be shown that urban stakeholders are essential mediators for transformation processes and retailers and that local online marketplaces can act as catalysts. That is useful against a background of planning and political communications processes for the legitimation of new transport infrastructures, as the individual groups of stakeholders begin to communicate, bringing about an increased awareness of their respective needs.

## Einzelhandel, Onlinemarktplätze und Radlogistik

**Abb. 1:**
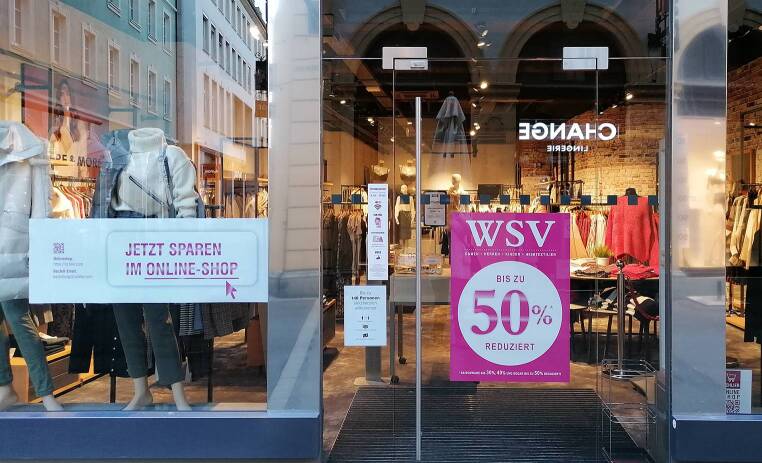
Stationäre Einzelhändler*innen in Würzburg reagieren auf die coronabedingten Lockdowns, u. a. indem sie den Onlineshop bewerben. (Foto: Februar 2021, eigene Aufnahme)

Kurier-Express-Paketdienste (KEP-Dienste) haben im Jahr 2020 über 4 Mrd. Sendungen (durchschnittlich 13 Mio. Sendungen pro Tag an ca. 8 Mio. Kund*innen) ausgeliefert, was einem Wachstum von 10,9 % im Vergleich zum Vorjahr entspricht (BIEK 2021, S. 6). Wichtigster Treiber für die Zunahme der Sendungen sind B2C(Business-to-Consumer)-Sendungen, die im Jahr 2020 um 18,6 % zugenommen haben (BIEK 2021, S. 6). Der starke Anstieg der B2C-Sendungen im Jahr 2020 kann zwar auf die Schließung stationärer Einzelhandelsgeschäfte während der coronabedingten Lockdowns (BIEK 2021, S. 12), und der damit einhergehenden Zunahme des Onlinehandels (HDE 2021; Abb. 1) zurückgeführt werden, bildet aber gleichzeitig den bereits in den Vorjahren identifizierten Trend der Steigerung von kleinteiligen Belieferungen von Privathaushalten ab (BMU und UBA 2019). Bei den coronabedingten Lockdowns kam es zu regional unterschiedlichen Einschränkungen des öffentlichen Lebens, die in Bayern besonders strikt ausfielen.

Empfänger*innen von KEP-Lieferungen sind vorwiegend der Handel (bis zu 8 Lieferungen täglich), die Gastronomie (bis zu 7 Lieferungen täglich) sowie private Endkund*innen (Agora Verkehrswende 2020, S. 16–17). Zudem ist davon auszugehen, dass sich aufgrund von Urbanisierungstendenzen, vielfältigeren E‑Commerce-Angeboten sowie veränderten Konsumgewohnheiten, der sich bereits abzeichnende Trend steigender B2C-Sendungen zukünftig weiter fortsetzt und die Verkehrsbelastung durch Lieferverkehre auch in Wohngebieten zunehmen wird (WEF 2020).

Um vor dem Hintergrund einer steigenden Anzahl an Sendungen das bereits im Jahr 2011 von der EU-Kommission (2011) in einem „Weißbuch zum Verkehr“ formulierte Ziel einer emissionsfreien Stadtlogistik bis 2030 zu erreichen, müssen neue Logistikkonzepte etabliert werden. Während im Rahmen zahlreicher Modellprojekte geeignete Alternativen getestet und u. a. Fahr- und Lastenräder als emissionsfreie und geeignete Vehikel für urbane Logistik identifiziert wurden, sind aktuelle Hemmnisse zur Steigerung der Nutzung von Radlogistik im Bereich des urbanen Warenwirtschaftsverkehrs die Verfügbarkeit von Radlogistik-Unternehmen an den jeweiligen Standorten (gerade in mittelgroßen Städten), die Bereitschaft von Logistikanbietern Fahr- und Lastenräder in die hauseigene Flotte zu integrieren, die Nutzung von Radlogistikanbietern durch ortsansässige Akteur*innen (Einzelhandel, Gastronomie) sowie fehlende Anreize von kommunaler/städtischer Seite zur Nutzung alternativer Lieferfahrzeuge (Agora Verkehrswende 2020; Gruber und Kihm 2016; Lenz und Riehle 2013; RLVD 2021).

Der vorliegende Beitrag thematisiert die Rolle von kommunalen/städtischen Akteur*innen und Einzelhändler*innen zur Etablierung emissionsfreier Liefermodi im städtischen Warenwirtschaftsverkehr anhand des Fallbeispiels *WüLivery*. *WüLivery* ist ein Same-Day-Delivery-Service für das Stadtgebiet von Würzburg, der in Kooperation des Stadtmarketingvereins *Würzburg macht Spaß*, der Wirtschaftsförderung der Stadt und dem Logistikunternehmen *Radboten* seit dem zweiten coronabedingten Lockdown im November 2020 in Würzburg angeboten wird.

Der stationäre Einzelhandel nimmt gerade in Innenstadtlagen eine zentrale Rolle hinsichtlich urbaner Logistik ein, da dieser einerseits Warenlieferungen empfängt und andererseits (zunehmend während der coronabedingten Lockdowns) Versender online bestellter Waren ist. „Die Innenstädte werden so gleichzeitig Ziel- und Startpunkt der letzten Meile zum Endverbraucher“ (Agora Verkehrswende 2020, S. 20). Die Lockdowns während der Covid-19-Pandemie haben darüber hinaus zu einer Zunahme von Multi- und Omnikanalstrategien im Einzelhandel geführt (Appel und Hardaker 2021) sowie zur verstärkten Etablierung von lokalen Onlinemarktplätzen, die Einzelhandels- und Logistikfunktionen verknüpfen. Während der coronabedingten Lockdowns konnten lokale Einzelhandelsunternehmen als Katalysator*innen für die Etablierung alternativer Stadtlogistikkonzepte eintreten. Obwohl sie ihre Ladenlokale zeitweise schließen mussten, konnten sie weiterhin über mediale Verkaufskanäle (z. B. Telefon, Mail, Onlineshop, Social Media) agieren und neue Lösungen zur Zustellung ihrer Güter an die Kund*innen aufbauen. Einerseits kann der Multi- und Omnikanalhandel als wesentlicher Treiber für die gesteigerte Güterverkehrsnachfrage im Bereich der KEP-Paketdienste identifiziert werden (Agora Verkehrswende 2020; Gruber 2020; WEF 2020), andererseits verfügen lokale Einzelhändler*innen und Onlinemarktplätze über die Entscheidungsmacht, mit welchen KEP-Dienstleistern und Kurierdiensten sie Vertragsverhältnisse eingehen (Agora Verkehrswende 2020, S. 21), und sind dadurch potenzielle Treiber zur Etablierung emissionsfreier Lieferverkehre auf lokaler Ebene.

Die vorliegende Arbeit hat zum Ziel, seit der Covid-19-Pandemie entstehende Möglichkeitsfenster zur Transformation bestehender Strukturen und Netzwerke in städtischen Räumen am Beispiel des Kooperationsprojekts *WüLivery* zu untersuchen. Der Fokus liegt auf der Rolle und Funktion von Einzelhändler*innen und lokalen Onlinemarktplätzen, die als potenzielle Katalysator*innen innerhalb städtischer Nachhaltigkeitstransformationen im Rahmen urbaner Logistik fungieren/auftreten. Damit leistet die Arbeit einen Beitrag zur Transformationsforschung urbaner Logistiksysteme. Im Rahmen dessen werden kommunale Akteur*innen (KA), Einzelhändler*innen (EZH), Fahrradlogistiker*innen (FL) und Betreiber*innen lokaler Onlinemarktplätze (LOM) zu der Belieferung von Kund*innen, zu Erfolgsfaktoren sowie zur damit verbundenen Restrukturierung des Kundenkontakts und der Rolle der lokalen Logistik (insbesondere bezogen auf *WüLivery*) für den Einzelhandel, befragt. Insgesamt wurden im Rahmen eines Forschungsprojekts zu den Auswirkungen der Covid-19-Pandemie auf den Einzelhandel (vorwiegend in der Würzburger Innenstadt) 34 Expert*inneninterviews seit Oktober 2020 durchgeführt. In der vorliegenden Analyse werden 11 Expert*inneninterviews berücksichtigt, in denen Radlogistik explizit angesprochen wurde.

Die Darstellung der empirischen Ergebnisse ist wie folgt gegliedert: Zuerst werden das Projekt *WüLivery* und der Logistikdienstleister *Radboten* vorgestellt. Die folgenden Unterkapitel stellen die Nutzung des Angebots durch Einzelhändler*innen dar, interpretieren das Verhältnis von Einzelhandel, lokalen Onlinemarktplätzen und urbaner Fahrradlogistik und zeigen die Rolle von Einzelhandel und lokalen Onlinemarktplätzen als Katalysator*innen für nachhaltigere urbane Logistik auf. Der Beitrag schließt mit einem Fazit.

## Fallbeispiel WüLivery

**Abb. 2:**
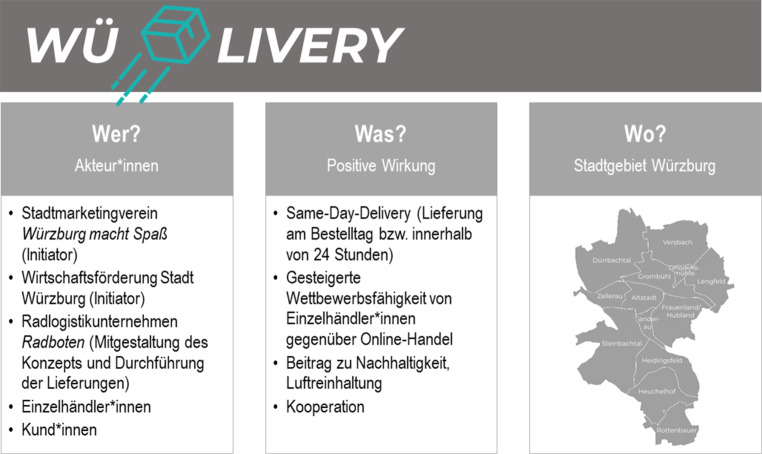
Akteur*innen, Wirkungen und Liefergebiet von *WüLivery*. (eigene Abbildung; Logo & Karte: *WüLivery* 2020)

*WüLivery* ist ein Same-Day-Lieferdienst, der vom Stadtmarketingverein *Würzburg macht Spaß* und der Wirtschaftsförderung der Stadt Würzburg initiiert wurde (Abb. 2). Der Lieferdienst startete im November 2020 im Zuge des zweiten coronabedingten Lockdowns. Die Idee zu *WüLivery* gab es bereits seit mehreren Jahren und war für die Umsetzung im Frühjahr 2020 geplant. Die coronabedingten Einschränkungen bremsten das Vorhaben im Frühjahr vorerst aus, wurden aber in der zweiten Jahreshälfte 2020 Anlass zur sofortigen Umsetzung des Konzepts (Interview XXXIV, KA). „… nachdem der erste Schock überstanden war, haben wir versucht Vollgas zu geben und das dieses Jahr noch umzusetzen“ (Interview XXIX, FL).

Die Idee orientierte sich ursprünglich an Shop-and-Drop-Modellen, wie beispielsweise in Augsburg, bei denen Kund*innen stationärer Einzelhandelsgeschäfte, ihre Adressdaten an der Kasse hinterlegen, ohne Einkaufstüten weiter durch die Stadt bummeln und die Einkäufe am gleichen Tag nach Hause geliefert bekommen.

Da ist die Idee gewesen, dass das ein Teil der Mobilitätswende in Augsburg war. Man wollte zu den Leuten sagen, kommt nicht mit den Pkws in die Stadt, kommt mit dem Fahrrad oder zu Fuß, kauft in der Innenstadt ein und lasst eure Pakete kostenlos nach Hause liefern (Interview XXXII, LOM).

**Abb. 3:**
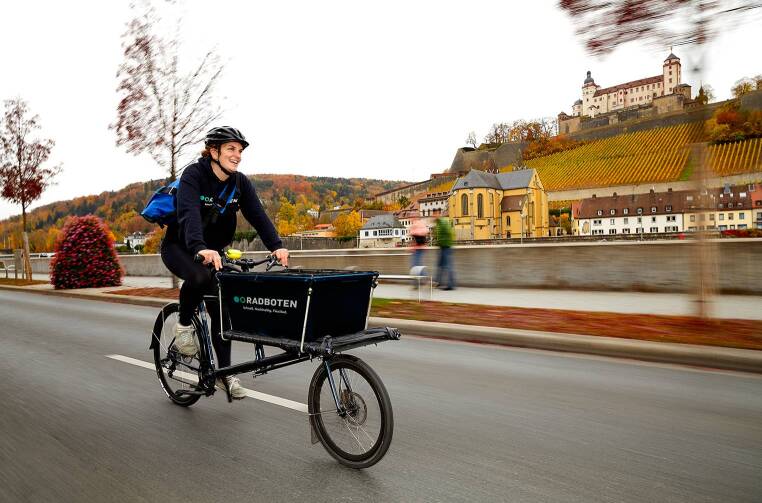
Lieferung per Lastenrad im Stadtgebiet von Würzburg durch die *Radboten*. (Foto: Benjamin Brückner)

In der Praxis weit häufiger genutzt wird allerdings die Option des „lokal online shoppen“ (WüLivery 2020), bei dem Würzburger Einzelhändler*innen, Kund*innen Same-Day-Lieferungen bis 19 Uhr im Würzburger Stadtgebiet anbieten können (Abb. 3).

### Logistikdienstleister: die Radboten

Mitkonzipiert und durchgeführt werden die Lieferungen von dem Würzburger Radlogistikunternehmen *Radboten*, aus deren Sicht ein stimmiges und funktionierendes Lieferkonzept für den stationären Einzelhandel in Würzburg vor allem unkompliziert und gut vermittelbar sein muss (Interview XXIX, FL).

Demnach wurden die Lieferpreise für das gesamte Stadtgebiet mit 4,50 € pro Lieferung vereinheitlicht. Fahrer*innen sollten keine Zahlvorgänge abwickeln müssen, da diese zeitaufwendig und fehleranfällig seien (Interview XXIX, FL). Zudem wurde die gesamte Organisation und Verwaltung digitalisiert, was als wichtiger Faktor benannt wird: „Bei uns läuft seit Anfang an wirklich fast alles digital. … Also ähnlich wie bei DHL, nur dass wir eben viel kleiner sind, noch“ (Interview XXIX, FL). Aufträge werden demnach online übermittelt und sind direkt einseh- und bearbeitbar, sodass auch die Kund*innen jederzeit wissen, wo sich ihre Lieferung befindet.

Die Kund*innen von *WüLivery* sind die Einzelhändler*innen, nicht die Endkund*innen: „Bei uns zahlen immer die Einzelhändler, weil die Einzelhändler auch entscheiden, wie viel [der Liefergebühren] sie an den Kunden weitergeben und wie viel sie selbst übernehmen“ (Interview XXIX, FL). Während der Pilotphase von *WüLivery* im November 2020 förderten die Wirtschaftsförderung und der Stadtmarketingverein der Stadt Würzburg die kompletten Lieferkosten von 4,50 € je Lieferung, sodass diese für die Einzelhändler*innen und Endkund*innen in den ersten Monaten entfielen. Seit Januar 2021 werden die Lieferkosten nur noch anteilig übernommen. Insgesamt übernahmen Stadtmarketing und Wirtschaftsförderung Kosten in Höhe von ca. 40.000,- € für umfassende Bewerbung des Projekts in Online- und Lokalmedien sowie Liefergebühren (Interview XXXIV, KA).

### Verstärkte Nutzung von WüLivery seitens des Einzelhandels

*WüLivery* wird seit dem ersten Tag stark genutzt, was auch auf den Zeitraum im Lockdown und vor Weihnachten zurückgeführt werden kann. Am Tag vor Weihnachten wurden 800 Sendungen im Würzburger Stadtgebiet ausgefahren. Das Unternehmen Radboten hatte zeitweise bis zu 9 Fahrer*innen gleichzeitig auf der Straße, während sie normalerweise mit 2–3 Fahrer*innen und 200 Sendungen pro Tag arbeiten. Bei einer Gesamtzahl von 583 Einzelhandelsunternehmen (Stand: 2020), nutzen im Oktober 2021 98 Händler*innen *WüLivery* nach wie vor regelmäßig (Interview XXXIV, KA). Eine Repräsentantin des Unternehmens *Radboten* weist darauf hin, dass *WüLivery* allein nicht profitabel wäre, sondern nur ein ergänzendes Produkt zu ihrem bereits bestehenden Logistikportfolio darstelle: „WüLivery würde alleine nicht funktionieren. … Das ist auch das Problem was viele andere Städte haben“ (Interview XXIX, FL).

Auch 2 andere Logistiker*innen und Betreiber*innen von lokalen Onlineplattformen in mittelgroßen bayerischen Städten bestätigen, dass Sendungen aus dem lokalen Einzelhandel i. d. R. nicht ausreichend sind, um Radlogistikdienstleistungen anzubieten. „Sich nur auf den stationären Einzelhandel zu konzentrieren, zu sagen ich bilde einfach die Fußgängerzone ab und nehme alle Händler auf die Plattform, das funktioniert nicht“ (Interview XXXIII, LOM). Während die Radboten neben *WüLivery* stark im Produktbereich klassischer Kurierdienste aufgestellt sind und bis Ende des Jahres 2019 95 % B2B (Business-to-Business)-Lieferungen im Bereich Büromaterial, Arzneimittel und Apothekenservice durchführten (Interview XXIX, FL), betreibt ein in Augsburg und Nürnberg operierendes Unternehmen, zusätzlich einen Onlinemarktplatz und ist stark fokussiert im Bereich der Essens- und Getränkelieferungen. Für *WüLivery* gibt es keinen gemeinsamen digitalen Marktplatz, sondern nur den Verweis auf die Kooperationspartner*innen im Einzelhandel in Würzburg auf der *WüLivery*-Homepage (WüLivery 2020). Ob die Lieferung durch *WüLivery* durchgeführt wird, entscheidet dann der/die Einzelhändler*in.

### Einzelhandel und Fahrradlogistik

Die Einzelhändler*innen nehmen im Rahmen von *WüLivery* fast schon eine paradoxe Position ein. Einerseits befürworten viele der interviewten Einzelhändler*innen Sendungen mittels der *Radboten* stark und stellen insbesondere den Aspekt der Nachhaltigkeit und Luftreinhaltung in den Vordergrund (z. B. Interviews XVIII, XXIII, EZH); andererseits sind es viele Einzelhändler*innen, die sich massiv gegen den Rückbau von Parkflächen und gegen Zufahrtsbeschränkungen der Innenstädte aussprechen, da sie von einem damit einhergehenden Rückgang der Kund*innenfrequenzen ausgehen. Ein Einzelhändler berichtet: „Wir haben im letzten Jahr immer wieder gehört, dass die Kunden aus dem Umland sagen „wir sind nicht mehr erwünscht“. … Es wurden über 500 Parkplätze vernichtet in den letzten ein bis zwei Jahren in Würzburg“ (Interview IV, EZH). Eine andere Händlerin sagt: „Autofrei ist irgendwie ganz schön, aber nur von schön kann der Einzelhandel nicht leben“ (Interview VIII, EZH). Zeitgleich kooperieren sie aber mit Radlogistiker*innen, die ganz andere Bedarfe im Hinblick auf eine Verkehrsinfrastruktur haben, nämlich u. a. ein durchgängiges gut befahrbares Radwegenetz mit wenig Konfliktpotenzial zwischen Rad‑, Fuß- und Autoverkehr. In stark verdichteten Städten, wie Würzburg, kann eine Radinfrastruktur aber nur auf Kosten von Park- und Straßenraum ausgebaut werden und bedarf der Legitimation durch Bevölkerung, ansässige Gewerbe und Handelsunternehmen sowie der kommunalen Politik (Interview XXXIV, KA).

*WüLivery* trägt dazu bei, die Sichtbarkeit und damit das Wissen über emissionsfreie Lieferverkehre in Würzburg zu erhöhen und wird vorwiegend positiv bewertet, sowohl von Einzelhändler*innen als auch von Kund*innen: „Die Kunden haben gesagt, wenn das damit auch funktioniert, ist das gut. Die musste man gar nicht überreden“ (Interview XVIII, EZH).

Obwohl es keine konkret negativen Bewertungen von *WüLivery* vonseiten der Interviewpartner*innen gab, gibt es trotzdem Einzelhändler*innen, die auch im Stadtgebiet Würzburg Sendungen aus organisatorischen Gründen selbst ausliefern oder per Post versenden (Interview XXV, EZH): „Dann waren es vielleicht einmal zwei Kunden für die ich das hätte nutzen können. Aber für die anderen zehn musste ich eh zur Post gehen, weil sie außerhalb von Würzburg leben. … Mir war das zu kompliziert.“

### Einzelhandel und lokale Onlinemarktplätze als Katalysator*innen für urbane Radlogistik

Insgesamt stellen Konzepte wie *WüLivery* und lokale Onlinemarktplätze Schnittstellenprojekte dar, in denen bisher wenig vernetzte Akteur*innen miteinander kooperieren und Veränderungen antreiben. Die durch Stadtmarketing und Wirtschaftsförderung initiierte Kooperation zwischen Radlogistik und Einzelhandel hat einen Kommunikationsprozess zwischen unterschiedlichen innenstadtrelevanten Akteur*innen in Gang gesetzt, bei dem das praktische Handeln in Form der Kooperation zwischen Einzelhandel und Fahrradlogistik bei der Warenlieferung am Anfang steht und durch die positive Erfahrung miteinander das Potenzial hat, mehr Verständnis für die unterschiedlichen Bedarfe zu generieren. Eine verstärkte Integration von Radlogistik für die Verteilung von Waren auf lokaler Ebene sowie geleichzeitige Digitalisierungsprozesse im stationären Einzelhandel können zumindest auf lokaler Ebene ein gesteigertes Bewusstsein für alternative Logistikmodi erzeugen (Interview XXIX, FL): „Ich denke bei manchen hat es dann schon Klick gemacht und man hat gesehen was möglich ist, wenn man das möchte, oder welchen Vorteil man dadurch haben kann.“

## Fazit

Die Ergebnisse zeigen, dass (1) die coronabedingten Lockdowns ein Möglichkeitsfenster zur Etablierung alternativer Strukturen in Teilbereichen der städtischen Logistik darstellen, (2) Einzelhändler*innen und lokale Onlinemarktplätze zu potenziellen Katalysator*innen im Prozess der Legitimation und Akzeptanz alternativer Liefermodi werden und (3) kommunale/städtische Akteur*innen eine grundlegende Rolle als Mediator*innen für die Etablierung nachhaltiger urbaner Verkehrssysteme einnehmen. Sie können durch die Unterstützung kooperativer Projekte Austausch und Kommunikation, die für Planung und Politik relevant sind, vorantreiben. Die Erkenntnisse der vorliegenden Studie sind dahingehend begrenzt, dass der Fokus vorwiegend auf einem Fallbeispiel liegt und die Bewertung der längerfristigen und zukünftigen Ausgestaltung der während der Coronapandemie umgesetzten Kooperationen weitere Erhebungen bedarf. Übergeordnet kann dennoch gesagt werden, dass Kooperationen von Akteur*innen aus unterschiedlichen Teilbereichen der städtischen Gesellschaft Bewusstsein schaffen für gegenseitige Bedarfe. Die coronabedingten Lockdowns stellen Gelegenheitsfenster dar, geplante Projekte zügiger umzusetzen, da viele Akteur*innen nach (neuen) Lösungen für einen konstruktiven Umgang mit der Pandemie und ihren Auswirkungen auf Städte und Einzelhandel suchen. *WüLivery* ist sowohl für das Unternehmen *Radboten* als auch für die Einzelhändler*innen eine Ergänzung und Diversifizierung des bereits bestehenden Produktportfolios. Für Planung und Politik ergeben sich daraus Ansatzpunkte zur Legitimation einer Veränderung und Transformation von urbanen Verkehrs- und Mobilitätssystemen, bei der autozentrierte Infrastrukturelemente zurückgebaut werden und Platz machen für nachhaltigere Systembausteine wie Fahrrad- und Fußgängerverkehr.
